# The apparent diffusion coefficient as a biomarker in the diagnosis of cervical cancer and the assessment of therapeutic response to chemoradiation therapy

**DOI:** 10.3389/fonc.2025.1610090

**Published:** 2025-09-09

**Authors:** Jelena Vasić, Nataša Prvulović Bunović, Milica Šarošković, Jelena Vuković, Stefan Stojanoski, Igor Nosek, Miloš Vuković

**Affiliations:** ^1^ Department of Radiology, Faculty of Medicine Novi Sad, University of Novi Sad, Novi Sad, Serbia; ^2^ Department for Radiology Diagnostics, Oncology Institute of Vojvodina, Sremska Kamenica, Serbia; ^3^ Department of Nuclear Medicine, Faculty of Medicine Novi Sad, University of Novi Sad, Novi Sad, Serbia; ^4^ Department of Gynecology and Obstetrics, Faculty of Medicine Novi Sad, University of Novi Sad, Novi Sad, Serbia; ^5^ Obstetrics and Gynecology Clinic, Clinical Center of Vojvodina, Novi Sad, Serbia

**Keywords:** DWI, cervical cancer, ADC, chemoradiation therapy, residual tumor, FIGO

## Abstract

**Introduction:**

The apparent diffusion coefficient (ADC) is a significant parameter in the diagnosis and monitoring of cervical cancer. The aim of this study is to evaluate ADC values in patients with cervical cancer, post-therapeutic changes, and normal findings, in order to assess their association with clinicopathological parameters, predict therapeutic outcomes, and differentiate residual tumors from post-treatment tissue without residual disease.

**Methods:**

A retrospective study included 148 patients divided into three groups: cervical cancer, post-therapeutic changes and normal findings. ADC values were measured by positioning ROI in the target tissue. Statistical analyses included ANOVA, t-tests, and ROC analysis.

**Results:**

The mean ADC values for cervical cancer (0.798 × 10^-3^ mm^2^/s) were significantly lower compared to post-therapeutic changes (1.394 × 10^-3^ mm^2^/s) and normal findings (1.431 × 10^-3^ mm^2^/s; p < 0.001). ADC values did not show statistically significant differences based on clinicopathological parameters. The change in ADC values after therapy (ΔADC: 0.607 × 10^-3^ mm^2^/s) indicated reduced cellularity. The mean ADC values of residual tumors (1.299 × 10^-3^ mm^2^/s) were significantly lower compared to post-therapeutic tissue without residual tumors (1.472 × 10^-3^ mm^2^/s; p = 0.029). The optimal value for distinguishing residual tumors from post-therapeutic tissue without residual tumors was 1.436 × 10^-3^ mm^2^/s. The optimal value for differentiating pre- and post-therapeutic tumor tissue was 0.929 × 10^-3^ mm^2^/s.

**Discussion:**

ADC proved to be a reliable imaging biomarker for differentiating cervical cancer, post-therapeutic changes, and normal findings, as well as for assessing therapeutic response. It demonstrated significant potential in distinguishing residual tumor tissue from post-treatment changes without residual disease.

## 1 Introduction

Cervical cancer remains one of the leading causes of cancer-related mortality in women, ranking second in incidence and third in mortality worldwide, with particularly high prevalence in developing countries. According to the World Health Organization, over 500,000 new cases are diagnosed annually, and approximately 250,000 women die from the disease (1). While preventive strategies such as HPV vaccination and organized screening programs have significantly reduced incidence in developed countries, cervical cancer remains a major public health challenge in resource-limited regions (2).

Concurrent chemoradiotherapy is the standard treatment for locally advanced cervical cancer (FIGO stage IIB-IVA), with five-year survival rates ranging from 75% for FIGO IB2 to 22% for FIGO IVA patients (2). Effective management depends on accurate disease staging, timely therapy initiation, and reliable treatment response assessment. Therefore, there is a critical need for early and robust prognostic biomarkers to guide therapeutic decision-making and improve patient outcomes (3).

Magnetic resonance imaging (MRI) plays a crucial role in non-invasive staging and monitoring due to its superior soft tissue contrast, aiding in FIGO staging assessment (3). Diffusion-weighted imaging (DWI) is a functional MRI technique sensitive to water molecule movement within tissues, providing insight into tumor microenvironment characteristics. The apparent diffusion coefficient (ADC), derived from DWI, quantitatively reflects extracellular water diffusion and cellular density (4, 5).

ADC measurement involves defining a region of interest (ROI) within the tumor and calculating its ADC values. Given the inverse relationship between ADC values and cellular density, this technique enables differentiation between malignant and benign tissues and allows precise monitoring of biological changes during therapy (6, 7). By detecting microscopic alterations preceding anatomical changes, ADC facilitates early assessment of treatment response and potential therapy adjustments. Multiple studies have confirmed the prognostic value of baseline ADC values and their changes throughout treatment. An increase in ADC during chemoradiotherapy reflects tumor cellularity reduction and effective treatment response, attributed to cell membrane breakdown and increased extracellular water diffusion (8–10). Conversely, low baseline ADC values correlate with more aggressive tumors, pronounced angiogenesis, and poorer outcomes (11).

Beyond its diagnostic role, ADC is emerging as a key prognostic biomarker. Longitudinal studies indicate that greater increases in intratumoral ADC during therapy are associated with prolonged overall survival and improved disease control (12). Additionally, pre-treatment minimum ADC values serve as independent predictors of local tumor control, time to progression, and cancer-specific survival (13). Integrating ADC metrics with clinical and radiological data supports the development of personalized treatment strategies, optimizing therapeutic efficacy while minimizing adverse effects.

This study investigates the role of ADC values in differentiating cervical cancer, post-therapeutic changes, and normal cervical findings. It evaluates treatment response by analyzing pre- and post-therapy ADC values and examines their correlation with clinical and pathological parameters, including FIGO stage, tumor grade, and lymph node metastases. Furthermore, this study evaluates the impact of these factors on post-therapeutic changes in ADC values, as well as the discriminatory potential of ADC in differentiating residual tumor from post-treatment tissue alterations.

## 2 Materials and methods

### 2.1 Subject selection

This retrospective study included 148 patients whose MRI scans were available in the database between 2013 and 2024. Patients were divided into three groups:

1. The first group consisted of 74 patients with a histopathologically confirmed diagnosis of squamous cell carcinoma of the cervix (following surgery or curettage);
2. The second group included 40 patients from the cervical cancer group but after undergoing chemoradiation therapy;
3. The third group comprised 74 control patients with normal cervical MRI findings.

The inclusion criteria for the first group were a histopathologically confirmed diagnosis of squamous cell carcinoma of the cervix (following surgery or curettage) and the availability of an initial diagnostic pelvic MRI in the database of the Oncology Institute of Vojvodina before any treatment (chemotherapy, radiation, or a combination). For the second group, the inclusion criterion was the availability of the first post-therapy MRI scan following chemoradiation. Patients in the control group were selected based on normal pelvic MRI findings and the absence of clinical or imaging indicators of gynecological pathology; subjects with benign abnormalities such as Nabothian cysts, uterine fibroids, or inflammatory conditions were excluded. The common inclusion criterion for all three groups was the presence of a DWI sequence with an ADC map as a standard part of the pelvic MRI protocol.

The exclusion criteria for the first group included pelvic MRI scans performed outside the Oncology Institute of Vojvodina, any prior therapy for cervical cancer before the MRI scan, and histological types other than squamous cell carcinoma.

The study was approved by the Ethics Committee of the Oncology Institute of Vojvodina (Approval No: 4/24/3-5228/2-4), and informed consent was not obtained due to its retrospective nature.

### 2.2 Patient data

As part of the research, data were retrieved from the information system of the Oncology Institute of Vojvodina, including tumor histology, differentiation grade, and FIGO stage.

### 2.3 Imaging analysis

MRI examinations were performed on two scanners: 1.5T (Siemens Aera, Erlangen, Germany) and 3T (Siemens Trio Tim, Erlangen, Germany). All patients underwent the following sequences: T1W, T2W, TIRM coronal tomograms, T1W parasagittal and T2W sagittal tomograms, T1W/T2W transverse tomograms, along with a DWI sequence with an ADC map in the transverse plane. To ensure consistency in quantitative analysis, both scanners employed an identical diffusion-weighted imaging protocol, using b-values of 0 and 800 s/mm^2^, a slice thickness of 4 mm, and standardized acquisition parameters.

ADC values for all three groups were measured using the PACS system (15). The DWI sequence was analyzed to define the tumor, which appeared as a high-intensity signal corresponding to the tumor mass location. The ROI was manually placed on the ADC map while simultaneously referencing other morphological MRI sequences to ensure accurate ROI placement on the primary tumor site. The ROI was positioned on the transverse section, encompassing the largest tumor area to obtain reliable ADC values that best reflect tumor cellularity. The precise localization of the tumor on post-therapy imaging and its differentiation from fibrotic tissue without tumor presence was performed through the analysis of the T2W sequence. Post-therapy residual tumor tissue typically appears as a hyperintense, nodular lesion with irregular margins, while a cervix altered by therapy without morphologically visible tumor presents as a hypointense, linear or diffuse area, reflecting fibrotic changes without evidence of mass effect. This determination is based on radiological criteria that distinguish viable tumor tissue from post-therapy fibrosis (16). For accuracy, all measurements were conducted by two independent readers in consensus.

Although the mean ADC values obtained from PACS measurements were initially expressed as (X̄ ± SD) × 10^-6^ mm^2^/s, the results were presented as (X̄ ± SD) × 10^-3^ mm^2^/s to align with the majority of available literature, facilitating easier comparison of obtained values.

### 2.4 Statistical analysis

Statistical analysis was performed using SPSS software version 27.0 (SPSS Inc, IBM, Armonk, NY), with results presented as mean ± standard deviation for normally distributed data and median (range) for non-normally distributed data. Normality was assessed using the Kolmogorov-Smirnov or Shapiro-Wilk test. Group comparisons were conducted using one-way ANOVA with *post-hoc* Tukey HSD tests for significant differences, while independent-samples t-tests were applied for binary comparisons. Although formal age-matching was not performed, a statistical comparison of age distributions across the three groups was conducted using the Kruskal–Wallis test to assess age comparability. Paired data were analyzed using the Wilcoxon signed-rank test. ADC variations based on FIGO stage and tumor grade were examined via ANOVA, while t-tests were used for binary clinical parameters, such as lymph node metastases and local tumor status. The impact of age, FIGO stage, and metastases on ADC changes was assessed through multiple linear regression. The discriminatory ability of ADC values for differentiating tumor residue from post-therapeutic tissue and pre- and post-therapy tumor tissue was evaluated using ROC analysis, with the Youden index applied to determine the optimal cut-off value, sensitivity, and specificity. To evaluate potential variability in ADC values due to MRI field strength, a sensitivity analysis comparing scans acquired on 1.5T versus 3T scanners was performed within the control group using the independent-samples t-test. A significance level of p < 0.05 was considered statistically significant.

## 3 Results

### 3.1 Demographic and histopathological data

This study included 148 patients divided into three groups: 74 patients with cervical cancer, 40 patients with post-therapeutically altered cervix, and 74 control patients with normal cervical findings. The mean age in the cervical cancer group was 56.17 ± 11.26 years, in the post-therapy group 57.30 ± 10.47 years, and in the control group 54.69 ± 16.60 years (**Table 1**). A comparison of age distributions between the three groups using the Kruskal–Wallis test showed no statistically significant difference (p = 0.887), confirming that the groups were comparable in terms of age. The characteristics of cervical cancer patients are presented in **Table 2**.

**Table 1:** Difference in mean ADC values between study groups.

Groups	N	Age (mean ± SD)	ADC (mean ± SD) x 10^-3^ mm^2^/s	P-value
< 0.001				
Cervical cancer	74	56.17 ± 11.26	0.798 ± 0.096	
Post-therapy altered cervix	40	57.30 ± 10.47	1.394 ± 0.252	
Normal cervix	74	54.69 ± 16.60	1.431 ± 0.182	

**Table 2:** Correlation between histopathological, clinical parameters and ADC values in patients with cervical cancer.

Parameters	N (%)	ADC (mean ± SD) x 10^-3^ mm^2^/s	P-value
Histological grade	37		0.247
Well differentiated (G1)	4 (10.81)	0.867 ± 0.099	
Moderately differentiated (G2)	30 (81.08)	0.779 ± 0.095	
Poorly differentiated (G3)	3 (8.11)	0.847 ± 0.261	
FIGO stage	74		0.708
I	11 (14.86)	0.815 ± 0.120	
II	20 (27.03)	0.777 ± 0.070	
III	35 (47.30)	0.801 ± 0.093	
IV	8 (10.81)	0.810 ± 0.138	
Local tumor status	74		0.620
Locally confined (I-IIA)	12 (16.22)	0.811 ± 0.115	
Locally advanced (IIB-IV)	62 (83.78)	0.795 ± 0.094	
Lymph nodes	74		0.854
Negative	34 (45.95)	0.800 ± 0.093	
Positive	40 (54.05)	0.796 ± 0.101	

### 3.2 Mean ADC values across study groups

**Figure 1:**
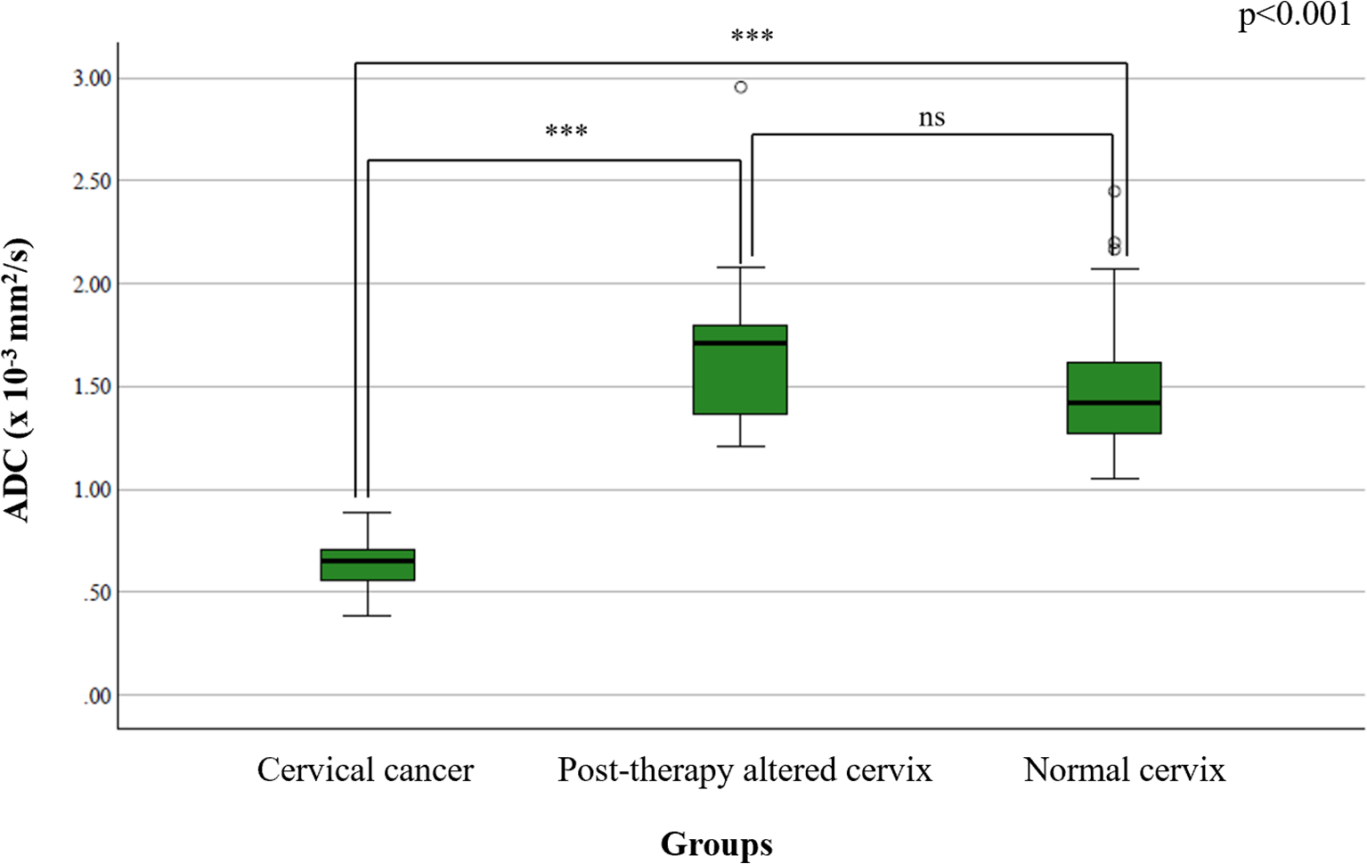
Comparison of mean ADC values between study groups – statistical distribution using box-and-whisker plots highlighting significant group differences. Statistical significance is indicated as follows: ns = not significant (p ≥ 0.05); ***p < 0.001.

**Figure 2:**
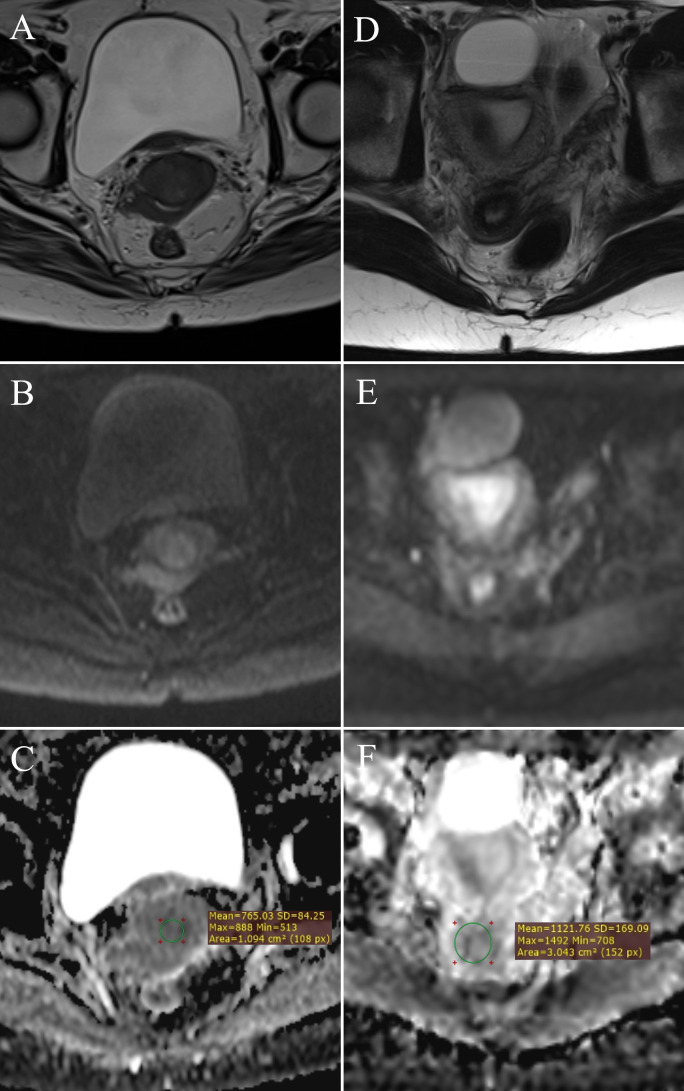
ADC value measurement: FIGO IIB stage cervical cancer – **(A)** (T2W), **(B)** (DWI), **(C)** (ADC map with ROI); normal cervix – **(D)** (T2W), **(E)** (DWI), **(F)** (ADC map with ROI).

The mean ADC value in the cervical cancer group was 0.798 ± 0.096 × 10^-3^ mm^2^/s, in the post-therapy group 1.394 ± 0.252 × 10^-3^ mm^2^/s, and in the control group 1.431 ± 0.182 × 10^-3^ mm^2^/s. One-way ANOVA revealed a significant difference among the groups (p < 0.001) (**Figure 1**). *Post-hoc* Tukey tests confirmed significant differences between the cervical cancer group and both the post-therapy (p < 0.001) and control groups (p < 0.001) (**Figure 2**), while no significant difference was found between the post-therapy and control groups (p = 0.534).

A sensitivity analysis was performed to assess whether magnetic field strength influenced ADC measurements. Within the control group, a comparison of ADC values between scans acquired on 1.5T and 3T scanners showed no statistically significant difference (p = 0.109), supporting the consistency of measurements across scanner types.

### 3.3 Analysis of mean ADC values based on clinical and pathological parameters

**Figure 3:**
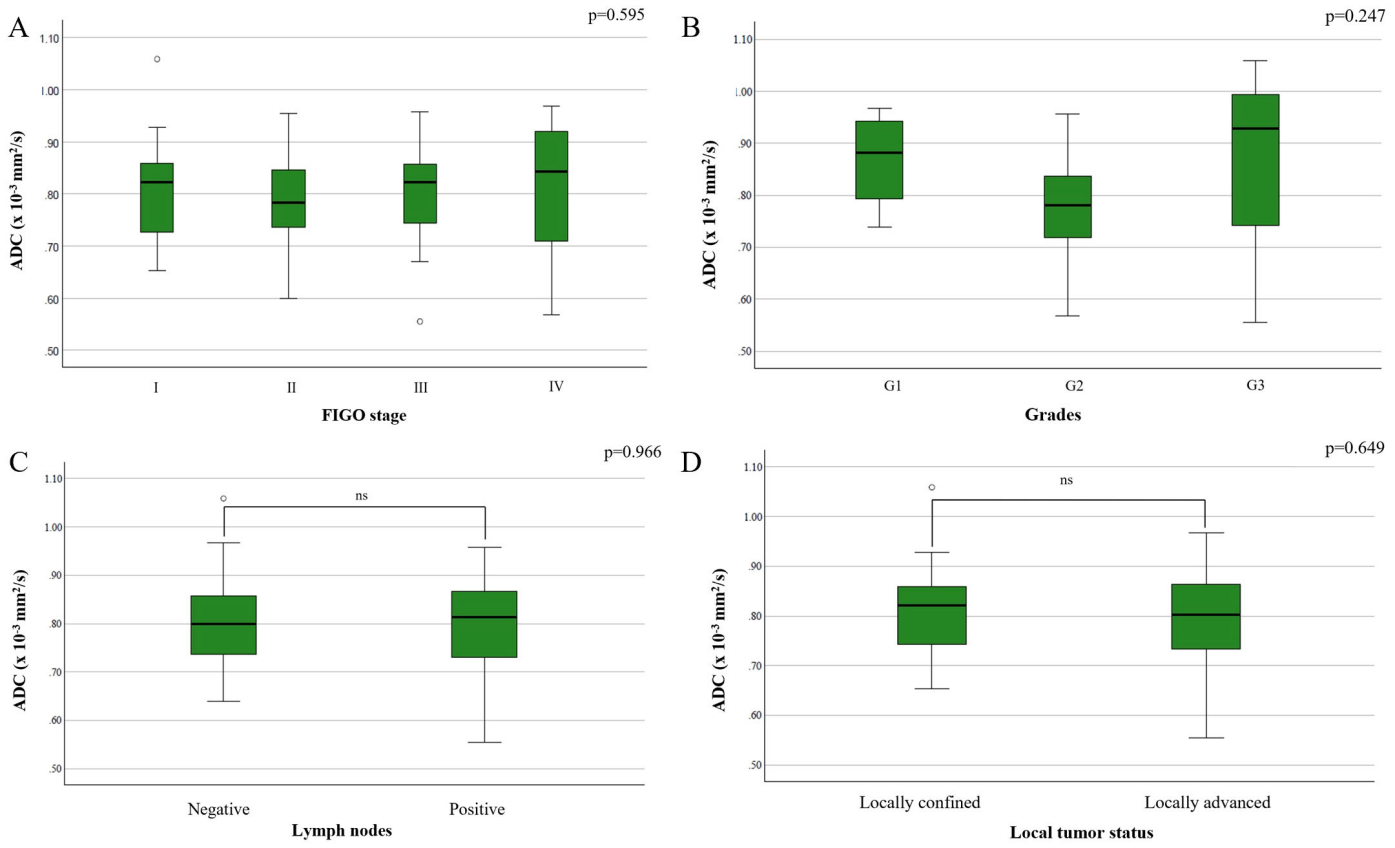
Comparison of mean ADC values depending on the: **(A)** FIGO stage; **(B)** different grades of cervical cancer; **(C)** presence of metastases in locoregional lymph nodes; **(D)** local tumor status.

There was no statistically significant difference in ADC values based on FIGO stage (F(3,70) = 0.465, p = 0.708) (**Figure 3A**), tumor grade (F(2,34) = 1.459, p = 0.247) (**Figure 3B**), lymph node metastases (p = 0.854) (**Figure 3C**), or local tumor status (p = 0.620) (**Figure 3D**).

### 3.4 Regression analysis of age, FIGO stage, and lymph node metastases on ADC changes

Multiple linear regression was performed to examine whether lymph node metastases, FIGO stage, and patient age predict ADC value changes. The model was not statistically significant (F(3,36) = 0.564, p = 0.642), explaining only R^2^ = 0.045 of the variance in ADC values. Neither lymph node metastases (B = −0.009, p = 0.794), age (B = 0.000, p = 0.824), nor FIGO stage (B = −0.009, p = 0.702) significantly influenced ADC changes. Multicollinearity analysis suggested potential overlap between FIGO stage and lymph node metastases (Tolerance = 0.228, VIF ≈ 4.4), contributing to the weak model significance.

### 3.5 Mean ADC values on post-therapy scans between patients with and without tumor residue

**Figure 4:**
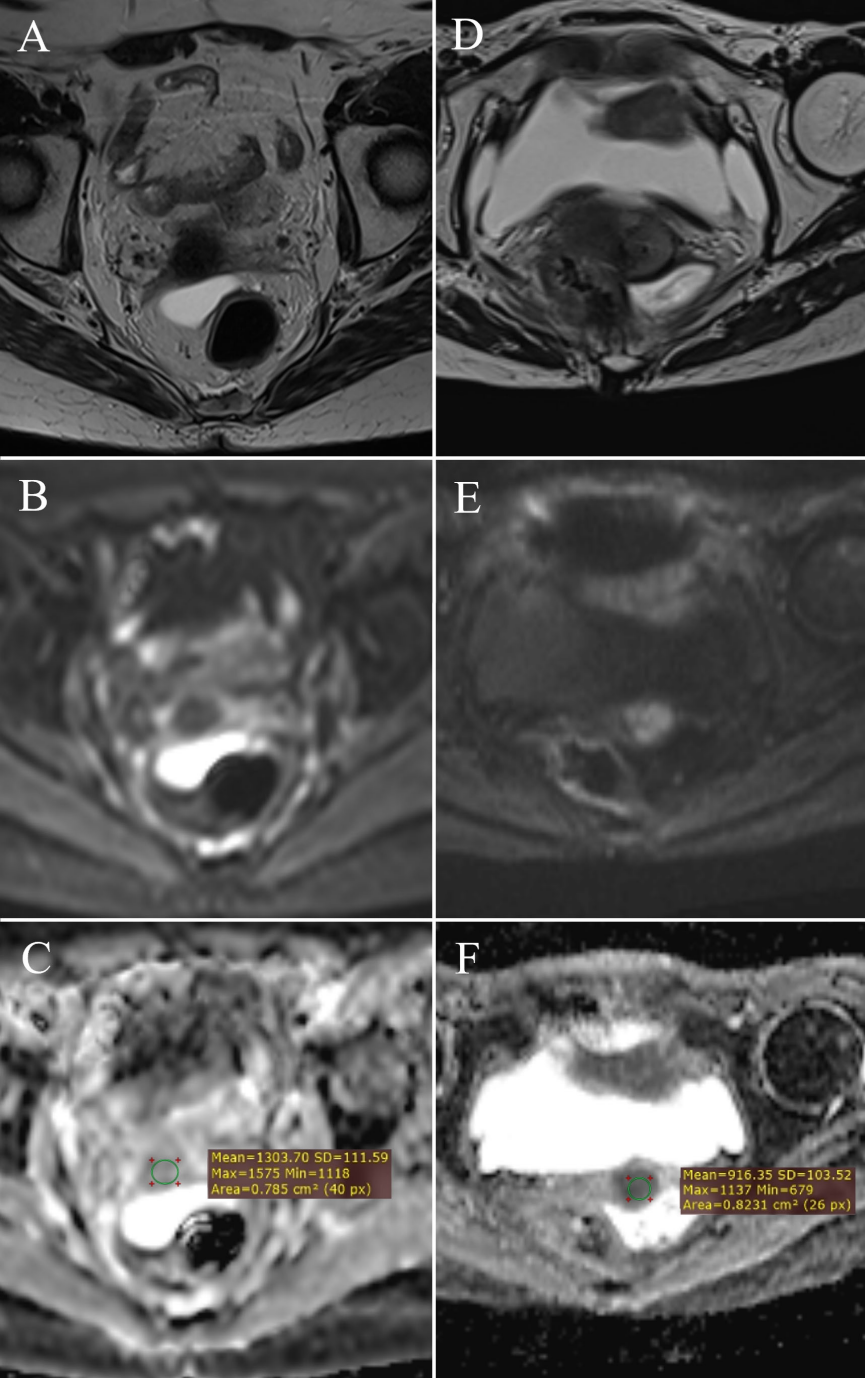
ADC value measurement: post-therapy cervix without tumor residue – **(A)** (T2W), **(B)** (DWI), **(C)** (ADC map with ROI); post-therapy cervix with tumor residue – **(D)** (T2W), **(E)** (DWI), **(F)** (ADC map with ROI).

The mean ADC value in patients with tumor residue on post-therapy MRI was 1.299 ± 0.267 × 10^-3^ mm^2^/s, while in those without residue, it was 1.472 ± 0.214 × 10^-3^ mm^2^/s. A significant difference was found between these groups (p = 0.029) (**Figure 4**).

### 3.6 Mean ADC values in tumor tissue before and after therapy

**Figure 5:**
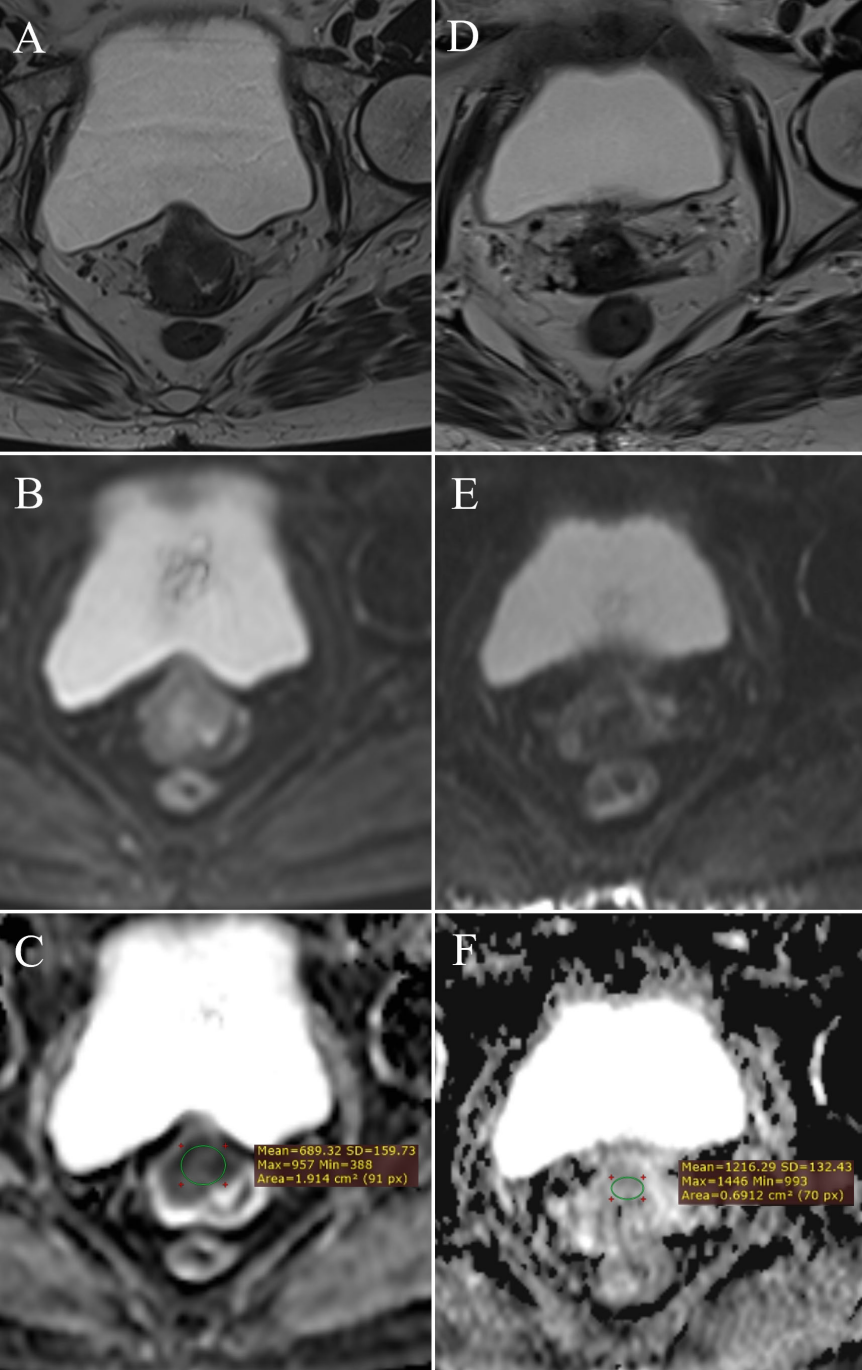
Change in ADC values: pre-therapy cervical cancer – **(A)** (T2W), **(B)** (DWI), **(C)** (ADC map with ROI); post-therapy cervical cancer – **(D)** (T2W), **(E)** (DWI), **(F)** (ADC map with ROI).

A significant difference was observed in ADC values between pre- and post-therapy tumor tissue (Z = -3.636, p < 0.001) (**Table 3**). The mean ADC value before therapy was 0.798 ± 0.096 × 10^-3^ mm^2^/s, increasing to 1.299 ± 0.267 × 10^-3^ mm^2^/s after therapy, indicating a reduction in tumor cellularity (**Figure 5**).

**Table 3:** Changes in ADC values of cervical cancer after therapy.

Subgroups	N	ADC (mean ± SD) x 10^-3^ mm^2^/s	P-value
< 0.001			
Cervical cancer before therapy	74	0.798 ± 0.096	
Post-therapy cervix with tumor residue	18	1.299 ± 0.267	
Post-therapy cervix without tumor residue	22	1.472 ± 0.214	

### 3.7 Effect of therapy on mean ADC values

In the cervical cancer group, the mean ADC value was 0.798 ± 0.096 × 10^-3^ mm^2^/s, significantly increasing to 1.394 ± 0.252 × 10^-3^ mm^2^/s post-therapy. The ADC change (ΔADC) between pre- and post-therapy measurements was 0.607 ± 0.042 × 10^-3^ mm^2^/s. These findings indicate a significant increase in ADC values following therapy, reflecting a reduction in tumor cellularity.

### 3.8 Determination of cut-off mean ADC values for group differentiation

**Figure 6:**
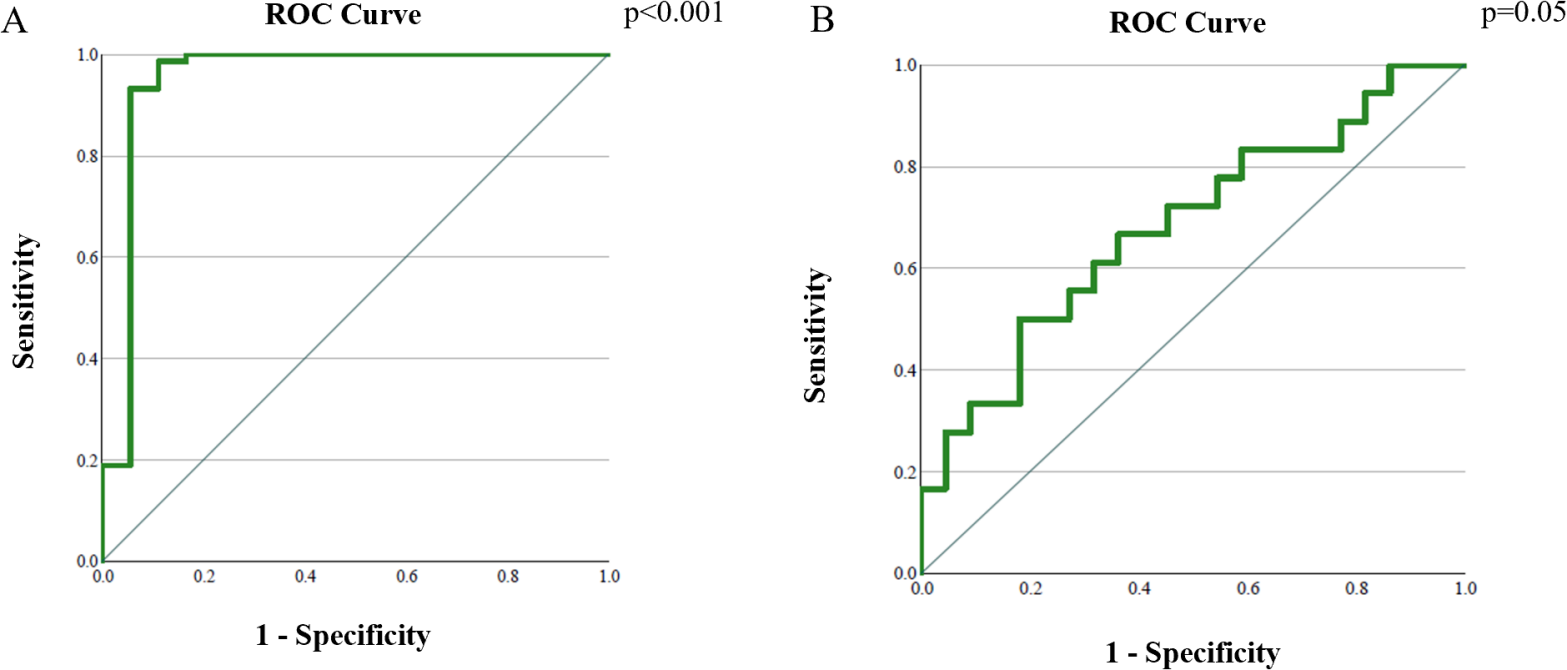
Analysis of the ROC curve of ADC values: **(A)** differentiation between cervical cancer tumor tissue before and after therapy; **(B)** differentiation between post-therapy altered cervix with and without tumor residue.

The area under the curve (AUC) for differentiating pre- and post-therapy tumor tissue was 0.95 (95% CI: 0.86–1.00, p < 0.001). The optimal ADC cut-off value was 0.929 × 10^-3^ mm^2^/s, with a sensitivity of 100% and specificity of 99.44% (**Figure 6A**, **Table 4**).

**Table 4:** Cut-off ADC values for differentiating the studied groups.

Groups	Cut-off ADC x 10^-3^ mm^2^/s	Sn^*^ (%)	Sp^*^ (%)	P-value
<0.001				
Cervical cancer/normal cervix	1.109	100	100	
Cervical cancer before/after therapy	0.929	100	99.4	
0.05				
Post-therapy cervix with/without tumor residue	1.436	66.7	63.6	

*Sn, sensitivity; Sp, Specificity.

The AUC for distinguishing tumor residue from post-therapeutically altered tissue without residue was 0.68 (95% CI: 0.51–0.85, p = 0.05). The optimal ADC cut-off value was 1.436 × 10^-3^ mm^2^/s, with a sensitivity of 66.7% and specificity of 63.6% (**Figure 6B**, **Table 4**).

## 4 Discussion

Cervical cancer has several well-established prognostic factors, including tumor size or volume, histological type and grade, FIGO stage, and lymph node status. However, since these variables have not proven to be adequate predictors of response to chemoradiation therapy, there is a growing need for a non-invasive biomarker that allows for a more detailed tumor characterization, enabling a personalized therapeutic approach and potentially improving clinical outcomes. Our findings confirm the value of ADC as a non-invasive imaging biomarker for distinguishing cervical cancer, post-therapeutic tissue alterations, and normal findings, as well as for detecting residual tumor following chemoradiation therapy.

The application of DWI has progressed from qualitative visual assessment to advanced quantitative analysis, enabling early prediction of tumor response to chemoradiation in cervical cancer. Consequently, ADC has emerged as a frequent subject of research and is gradually being integrated into clinical practice.

Our study demonstrates significantly lower ADC values in cervical cancer patients compared to post-therapeutically altered tissue and normal findings, consistent with previous studies that identify low ADC values as characteristic of malignant tissues due to their high cellularity and restricted water molecule movement (2, 7).

In our analysis, post-therapy tissue with residual tumor showed lower ADC values compared to post-therapy tissue without residue, highlighting the ability of ADC to differentiate residual tumor cells from regenerative or fibrotic tissue. However, it is important to acknowledge that the observed sensitivity (66.7%) and specificity (63.6%) reflect only moderate diagnostic accuracy. These values, while suggestive, are insufficient for standalone clinical decision-making. Several studies have indicated that ADC changes during chemoradiation can predict clinical response in cervical cancer patients (12–15, 17). Similarly, Schreuder et al. demonstrated the effectiveness of ADC in monitoring the response to chemoradiation in cervical cancer patients (7).

ROC analysis in our study confirmed the high diagnostic accuracy of ADC in distinguishing tumor tissue before and after therapy. Similarly, Yin et al. conducted a related analysis and successfully differentiated pre- and post-therapy tumor tissue using comparable techniques, further supporting the effectiveness of this approach (4).

Beyond its diagnostic role, ADC also holds prognostic potential. Our results demonstrate a significant post-therapy increase in ADC values, reflecting reduced tumor cellularity and indicating potential prognostic value for long-term outcomes. Moreover, the observed similarity in ADC values between the post-therapy and control groups is clinically plausible, as fibrotic changes following effective chemoradiation, in the absence of residual tumor, may resemble the diffusion characteristics of normal cervical stroma. Holopainen et al. reported that greater increases in intratumoral ADC values post-therapy correlate with improved overall survival in cervical cancer patients (9). Bae et al. further emphasized that changes between pre-therapy ADC values and mid-treatment measurements can accurately predict tumor recurrence, highlighting their potential in clinical follow-up (8). Nevertheless, only a limited number of studies with sufficiently large patient cohorts have investigated the prognostic value of baseline ADC in predicting long-term survival and tumor recurrence (18–21). Given the low coefficient of determination (R^2^ = 0.045), the predictive value of our regression model examining the relationship between ADC values and clinical outcomes is limited.

In our study, ADC values did not show statistically significant differences based on FIGO stage, tumor grade, lymph node metastases, or local tumor status. This suggests that the reduction in cellularity is a universal biological response to therapy, independent of clinical-pathological characteristics. While FIGO stage remains a key prognostic factor for cervical cancer survival, our findings are consistent with those of Bae et al., who also reported that changes in ADC values are not significantly associated with FIGO stage (8). Tumor heterogeneity and the use of single-slice ROI methodology may serve as plausible explanations for the absence of correlation between ADC values and clinical-pathological features, as these factors may inadequately represent tumor biology in its entirety. In contrast, Nakamura et al. reported that pre-therapy ADC values correlate significantly with FIGO stage, tumor size, parametrial invasion, and lymph node metastases (18). These discrepancies likely arise from non-standardized methods for calculating and presenting ADC values.

This study has several limitations. First, its retrospective design and limited follow-up period prevented us from assessing the potential impact of ADC on long-term survival. Further prospective studies are needed to obtain these data. Although MRI scans were acquired using both 1.5T and 3T scanners, a sensitivity analysis in the control group showed no significant difference in ADC values between scanner types (p = 0.109). This supports the consistency of measurements across field strengths under standardized imaging protocols. Additionally, a study by Caruso et al. (22) similarly found no statistically significant ADC differences between scanners of different field strengths, reinforcing the reliability of pooled ADC data. One of the most significant limitations is the relatively small number of patients in certain subgroups, particularly those with post-therapy MRI scans, as well as the lack of standardization in ADC measurements. Due to the limited size of the post-therapy subgroup with residual tumor, the ROC analysis had moderate statistical power (≈0.65). Although the result reached significance (AUC = 0.68, p = 0.05), further validation on larger cohorts is warranted. Increasing the patient sample and using whole-tumor volume measurements instead of a single-slice method could enhance the study’s scope. One of the limitations of this study is the absence of post-therapy PET/CT scans in patients who were radiologically classified as having a tumor residue. PET/CT would allow for the assessment of metabolic activity, providing additional diagnostic confidence in differentiating viable tumor tissue from fibrotic and inflammatory post-therapy changes. Its inclusion in the evaluation would offer more precise information regarding the presence of tumor residue and potentially reduce the risk of false-positive or false-negative MRI findings.

Our study confirms that ADC is a highly reliable diagnostic parameter for cervical cancer, enabling precise differentiation between tumor tissue, post-therapy changes, and normal tissue. To our knowledge, this is the first study in the available literature to establish the discriminative ability and a clear cut-off ADC value for distinguishing tumor residue from post-therapeutic fibrotic tissue without residue. A particularly important finding is the increase in ADC values after therapy, reflecting reduced tumor cellularity and reinforcing its role as a prognostic biomarker for assessing chemoradiation therapy success. These results strongly support the integration of ADC into clinical practice to enhance diagnostic accuracy and personalize therapeutic strategies. To enhance clinical utility, future studies should incorporate advanced imaging analytics, including diffusion-weighted texture analysis and radiomics, which may improve diagnostic precision in distinguishing viable tumor tissue from post-treatment fibrosis.

ADC values serve as a useful biomarker for distinguishing cervical cancer from post-therapeutic and normal tissue. Lower ADC values in tumors indicate increased cellularity, aiding in malignancy detection and differentiation from benign changes. Additionally, ADC effectively identifies residual tumors, providing a non-invasive tool for assessing treatment response. However, ADC variations did not correlate with clinical and pathological parameters such as tumor stage, grade, or lymph node involvement, suggesting a consistent biological response to therapy. These findings highlight the potential of ADC in improving diagnostic accuracy and treatment evaluation in cervical cancer.

## Data Availability

The raw data supporting the conclusions of this article will be made available by the authors, without undue reservation.
